# 15-year patient-reported outcomes of a cemented flanged cup and stem combination in primary total hip arthroplasty: a New Zealand study

**DOI:** 10.1177/11207000251371132

**Published:** 2025-10-31

**Authors:** Amy Pearce, Chaitanya Joshi, Georgina Chan, Tony Lamberton, Simon MacLean, Andrew Vane, Kim Hébert-Losier

**Affiliations:** 1 University of Waikato, Te Huataki Waiora - School of Health, Hamilton, Waikato, New Zealand; 2 Waikato Institute of Technology, Centre for Sport Science and Human Performance, Hamilton, Waikato, New Zealand; 3 University of Auckland, Department of Statistics, Auckland, New Zealand; 4 Tauranga Hospital, Tauranga, Bay of Plenty, New Zealand; 5 Grace Orthopaedic Centre, Tauranga, Bay of Plenty, New Zealand

**Keywords:** Oxford Hip Score, postoperative, preoperative, VR-12, WOMAC

## Abstract

**Methods:**

We investigated 15-year patient-reported outcomes (PROMs) and their predictors in primary total hip arthroplasty (THA) for osteoarthritis using a cemented flanged cup and stem from a regional joint registry in New Zealand. Regional data were collected for all primary THAs with this cemented combination from 1 January 2003 to 30 June 2023 who had recorded PROMs on at least 1 occasion (*n* = 263). PROMs included Oxford Hip Score, Western Ontario and McMaster Universities Arthritis Index and Veterans Rand-12, evaluated against patient age, ethnicity, sex, body mass index (BMI), funding pathway, and American Society of Anesthesiologists (ASA) rating.

**Results:**

Significant improvements across preoperative PROMs were noted 1-year post-surgery, with a mean change above 23 in the Oxford Hip Score maintained at 5, 10, and 15 years (*p* ⩽ 0.001).

**Conclusions:**

Regression analysis indicated that being female, public funding, and higher BMI were associated with worse preoperative PROMs. Poorer preoperative scores, older age and ASA 3 rating correlated with poorer postoperative outcomes.

## Introduction

Surgical success for joint replacement procedures has long been evaluated using implant survivorship models.^1^ With current healthcare shifting focus to patient well-being and quality of life,^2^ patient-reported outcome measures (PROMs) have become an important metric in quantifying surgical success. PROMs allow surgeons to quantify patients’ health-related quality of life and make decisions regarding optimal patient care, shifting the focus from surgeon-centred to patient-centred models of care.

The cemented, all-polyethylene flanged cup we chose to examine in this study, the Exeter Contemporary Flanged cup (Stryker Orthopaedics, Mahwah, NJ, USA), has been in use since 1991 and was the cemented cup most used in New Zealand (NZ) between 2003 and 2013.^3,4^ Schmitz et al.^5^ reported good results for this cup in THA patients under 50 years with a component survival rate of 90% after 10 years. Furthermore, Maggs et al.^6^ identified a 97.8% survivorship at 12.5 years in patients 46 years and above. In environments where THAs are completed in high volumes, the polished, stainless steel, tapered, and collarless cemented stem, the Exeter V40 stem (Stryker Orthopaedics) we are examining in this study, has demonstrated excellent functional outcomes based Western Ontario and McMaster Universities Arthritis Index (WOMAC) scores and 97.8% survivorship at 10 years.^7,8^ This stem has a reported 15-year survivorship of 96.6% (confidence interval [CI] 95%, 98.3–94.8) and rare periprosthetic fracture rates of 0.6% (CI 95%, 0.3–0.9) at 15 years,^9^ although this may be underestimated in studies where periprosthetic fractures requiring alternative reoperations (such as open reduction and internal fixation, and where there is no stem exchange),^10–12^ have not been reported. In NZ, over 54,000 of these particular stems were used between 1999 and 2022 and currently represent 60% of all stems used in the UK.^4,8^ This cemented combination maintained a top 10 position of all hip combinations implanted in NZ between 2003 and 2013.

Although studies have reported survivorship of this Exeter Contemporary Flanged cup with Exeter V40 stem (Stryker Orthopaedics) combination,^6^ few have examined PROMs. A more thorough investigation of this cemented cup-and-stem combination specific to osteoarthritis that includes short-, medium- and long-term PROMs evaluated against patient characteristics, comorbidities, and funding pathways may identify specific predictors affecting PROMs and populations susceptible to poorer outcomes.

Prospectively gathered data from a regional registry were used to examine preoperative, 1-year, 5-year, 10-year, and 15-year PROMs in primary THA for osteoarthritis with this cup-and-stem cemented combination against patient characteristics age, sex, and body mass index (BMI); comorbidities based on American Society of Anesthesiologists (ASA) rating; and funding pathways. Oxford Hip,^13^ WOMAC, and health-related quality of life Veterans Rand 12-item (VR-12)^14^ scores were analysed. The VR-12 has been validated by cross-system comparisons.^15^

We aimed to report PROMs for this implant combination up to 15 years, establish a benchmark methodology for future studies of other implant combinations in this region, and determine predictors of these PROMs to guide THA management in this region of interest.

## Method

### Data source and collection

Tauranga Orthopaedic Research (TOR) is a non-profit organisation in the Bay of Plenty region of NZ which maintains a mandatory registry of all regional joint arthroplasty surgeries. The TOR registry is independent of the NZ National Joint Registry and includes procedures from 2 public and 1 private hospital. Data are collected with patient consent and entered into the Standardized Orthopaedic Cartilage Repair and Treatment Evaluation Software (SOCRATES version 3.5.8.26 10150).

The TOR registry collects Oxford, WOMAC, and VR-12 as their routine standard of care for THA preoperatively, at 1 year postoperatively, at 5 years postoperatively, and every 5 years thereafter. The University of Waikato’s Human Research Ethics Committee granted ethical approval to analyse the database for this study (HREC2023#12).

**Figure 1.**
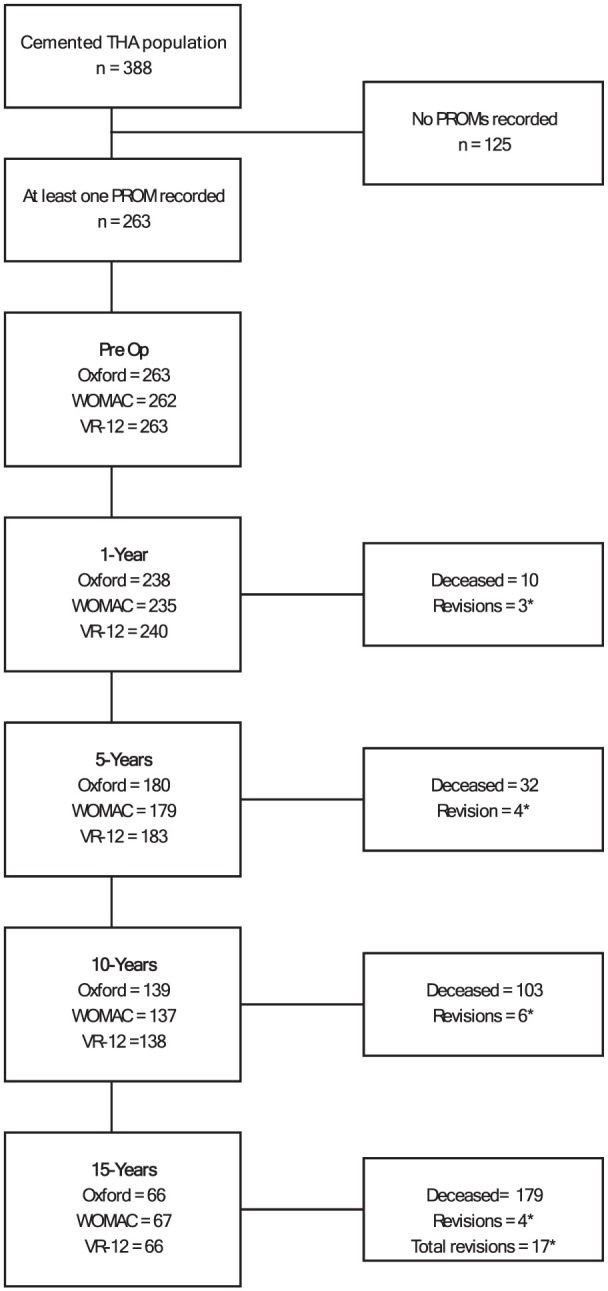
Flow chart depicting sample sizes, deaths and revisions at each follow-up period. All unaccounted observations were those lost to follow-up (questionnaires not returned, incomplete questionnaires or withdrawal from the study at that timepoint). *See Supplemental Table 2 for detailed reasons for revision and revision type.

Anonymised prospective observational data from 1 January 2003 to 30 June 2023 were retrieved in July 2023. Data were eligible for analysis when patients underwent primary THA for osteoarthritis and received an Exeter Contemporary Flanged cup with Exeter V40 stem combination within the set observational period. Patient data needed to contain at least 1 of the PROMs at any of the timepoints of interest to meet inclusion. A pairwise deletion approach was implemented to handle missing PROM scores at any timepoint (questionnaires not returned, incomplete questionnaires or withdrawal from the study at that timepoint) so that all available data were used in statistical analysis (Figure 1).^16^

### Outcomes

We considered preoperative PROM data as scores recorded at any point preoperatively; 1-year data as scores recorded from 10–14 months postoperatively; 5-year data as scores recorded from 4–6 years postoperatively; 10-year data as scores recorded from 9–11 years postoperatively; and 15-year data as scores recorded from 14–16 years postoperatively. Although 15-year data were available for some patients, not all measures of interest had observations. Consequently, comparison of PROMs up to 10 years postoperatively were included in subgroup analysis.

### Predictors

The predictors extracted from the database included patient age, sex, BMI, ethnicity, ASA rating, and funding pathway. Age and BMI were treated as continuous variables. Other predictors were treated as categorical or rank variables, and included sex (female, male), funding pathway (public, private), ethnicity (NZ European, Māori, Other), and ASA rating (a single ASA 4 observation was recoded as ASA 3 for analysis). “Other” ethnicity included “undetermined”, “undisclosed”, and any ethnicity not recorded as Māori or NZ European.

### Data analysis

Data were inspected and normality of distribution verified. Descriptive statistics were computed for all variables, including counts, means, standard deviations (SD), and ranges (minimum, maximum). Continuous numeric dependent variables were compared using 2-sample *t*-tests when normally distributed. Normal sampling mean distribution was assumed in samples ⩾30 according to central limit theorem given parametric tests produce more accurate and precise estimates with higher statistical powers.^17^ Where variances were unequal, Welch 2-sample *t*-tests were performed. Analysis of variance (ANOVA) tests were performed to compare predictors with more than 2 categories, dependent on ANOVA assumptions being met.^18^ The Tukey method was used in *post-hoc* testing.

Multiple linear regression analyses were performed on preoperative and postoperative PROMs to determine if any of the predictors correlated to any of the PROMs. The Bayesian Information Criterion was used to determine best model fit.^19^ Estimates and coefficients of determination (*R*^2^) were extracted from the regressions to quantify the association between predictors and outcomes, as were standard errors to quantify uncertainty of estimates. Possible predictors of preoperative scores included age, ethnicity, sex, funding pathway, BMI, and ASA rating. Postoperative predictors included the same factors, but also preoperative scores as these have been identified as possible predictors of postoperative scores.^20^ Collinearity was investigated with variance inflation factor and condition index. Significance levels were set to *p* < 0.05 and confidence intervals to 95% (lower, upper). All statistical tests were performed using RStudio 2023.09.1 build 494 “Desert Sunflower” statistical software.^21^

## Results

A total of 388 THAs met inclusion for the defined period. Observations that did not have at least 1 PROM recorded were excluded, leaving 263 THAs for analysis (Table 1), with follow-up rates of 91%, 68%, 53%, and 26% at 1, 5, 10, and 15 years, respectively. Of the 263 observations with at least 1 PROM recorded, females accounted for 54.3% (*n* = 143), ASA 2 rated patients for 62% (n = 164) and NZ Europeans for 92.1% (*n* = 242). The mean age of patients at surgery was 71.4 years (range 46–92) and mean BMI 28.01 kg/m^2^ (range 18.2–50.4). Five males and seven females received bilateral THAs. The standard 150-mm stem length accounted for 92.3% of all stems implanted, with 125-mm accounting for 4.6% and 205-mm at 0.7%. Stem length was not recorded in 2.4% of observations. No statistically significant differences in mean age, BMI or factor proportions between the total sample of 388 and the evaluated 263 observations were noted (Supplemental Table 1). Seventeen revisions during the observation period were noted. (Supplemental Table 2).

**Table 1.** Descriptive statistics of preoperative patient characteristics for observations with at least 1 patient-reported outcome measure.

		Sex		Funding pathway		Ethnicity		
Variable	Sample *n*= 263	Male *n* = 120 (45.6%)	Female *n* = 143 (54.4%)	Public *n* = 87 (33.1%)	Private *n* = 176 (66.9%)	NZEU *n* = 242 (92.1%)	Māori *n* = 9 (3.4%)	Other *n* = 12 (4.5%)
Age^a^	71.38 ± 8.67	70.84 ± 8.35	71.83 ± 8.94	71.75 ± 8.78	71.20 ± 8.64	71.90 ± 8.40	66.00 ± 8.47	64.83 ± 10.87
BMI^a^	28.01 ± 4.75	28.14 ± 3.57	27.90 ± 5.58	28.68 ± 4.24	27.75 ± 4.92	27.95 ± 4.84	30.95 ± 2.54	27.62 ± 3.77
ASA Grade^b^								
I	49 (19%)	22 (18%)	27 (19%)	13 (15%)	36 (20.5%)	46 (19%)	2 (2.2%)	1 (8.3%)
II	164 (62%)	72 (60%)	93 (65%)	46 (52.5%)	118 (67%)	151 (62%)	3 (3.3%)	10 (83.4%)
III	46 (18%)	26 (22%)	20 (14%)	24 (28%)	22 (12.5%)	41 (17%)	4 (4.5%)	1 (8.3%)
IV	−	−	−	−	−	−	−	−
Not recorded	4 (2%)	1 (0%)	3 (2%)	4 (3.5%)	−	4 (2%)	−	−

ASA, American Society of Anesthesiologists Physical Status classification; BMI, body mass index; NZEU, New Zealand European.

a The values are given as the mean and the standard deviations.

b Values are given as the number of patients, with percentage (number of patients divided by the total) in parentheses. Due to missing values, some categories do not equal the total number of patients for each column.

### Patient-reported outcome measures

Descriptive statistics of PROMs, including the subscales of both WOMAC and VR-12, are reported in Table 1 for preoperative, 1-, 5-, 10-, and 15-year timepoints. 1-way ANOVA revealed statistically significant differences in mean Oxford, WOMAC, and VR-12 scores between at least 2 timepoints. *Post-hoc* testing revealed preoperative PROMs were statistically significantly different to their respective 1-, 5-, 10-and 15-year postoperative PROMs. There were no significant differences in postoperative PROMs between their respective 1-, 5-, 10-, and 15-year timepoints. Mean postoperative VR-12-physical scores for the sample were worse with scores less than the standardised T-score metric mean of 50 (Standard deviation = 10) across timepoints. In contrast, mean postoperative VR-12-mental scores were better, with scores greater than the standardised score of 50.

All mean preoperative PROMs were statistically significantly poorer in females than males (*p* ⩽ 0.041) (Table 2). Postoperatively, WOMAC-total and function scores for females were also statistically significantly poorer 1-year and 5 years postoperatively, as were WOMAC-motion and VR-12-physical scores 1-year postoperatively. Males and females showed no statistically significant difference in postoperative Oxford, WOMAC-pain, or VR-12-mental scores at 1, 5, and 10 years postoperatively.

**Table 2.** Descriptive statistics of patient-reported outcomes.

PROM	Sample	Mean ± SD	Range	ANOVA	Mean diff (95% CI)	*Post hoc*
	(*n*)		(min–max)	*(p-*value)		*(p-*value)
**Oxford (0–48)** ^a^						
Pre-op	263	17.75 ± 8.73	2–47	<0.001*		
1-year	238	41.30 ± 7.06	8–48		−23.38 (−25.35–−21.42)	<0.001*
5-year	180	41.62 ± 7.48	12–48		−0.49 (−2.66–1.68)	0.970
10-year	139	41.30 ± 8.46	9–48		−0.13 (−2.64–2.39)	0.990
15-year	66	39.44 ± 9.58	13–48		−2.05 (−5.36–1.26)	0.440
**WOMAC total (0–96)** ^b^						
Pre-op	262	54.82 ± 19.27	0–96	<0.001*		
1-year	235	12.36 ± 12.68	0–67		42.46 (38.41–46.52)	<0.001*
5-year	179	13.18 ± 14.47	0–68		0.82 (−3.66–5.30)	0.987
10-year	137	14.66 ± 16.32	0–78		1.47 (−3.65–6.60)	0.935
15-year	67	18.91 ± 21.01	0–89		−4.25 (−10.98–2.48)	0.417
**WOMAC pain (0–20)** ^b^						
Pre-op	262	11.02 ± 4.01	0–20	<0.001*		
1-year	235	1.69 ± 2.49	0–14		9.33 (8.49–10.16)	<0.001*
5-year	180	1.87 ± 3.04	0–15		0.17 (−0.76–1.09)	0.990
10-year	139	2.45 ± 3.58	0–14		0.59 (−0.47–1.64)	0.550
15-year	67	3.15 ± 4.24	0–18		−0.70 (−2.09–0.69)	0.650
**WOMAC function (0–68)** ^b^						
Pre-op	262	39.05 ± 14.35	29–68	<0.001*		
1-year	235	9.30 ± 9.68	0–48		29.74 (26.71–32.78)	<0.001*
5-year	180	10.00 ± 11.20	0–53		0.70 (−2.64–4.05)	0.980
10-year	138	10.88 ± 12.07	0–58		0.87 (−2.95–4.69)	0.970
15-year	67	13.87 ± 15.39	0–63		−2.99 (−8.02–2.04)	0.480
**WOMAC motion (0–8)** ^b^						
Pre-op	262	4.76 ± 1.71	0–8	<0.001*		
1-year	235	1.36 ± 1.43	0–6		3.39 (3.01–3.78)	<0.001*
5-year	179	1.25 ± 1.39	0–6		−0.12 (−0.54–0.31)	0.950
10-year	137	1.35 ± 1.56	0–6		0.11 (−0.38–0.59)	0.980
15-year	67	1.90 ± 1.96	0–8		−0.55 (−1.19–0.09)	0.140
**VR-12 physical ( $\bar{x}$ = 50)** ^c^						
Pre-op	263	26.52 ± 8.22	8.5–54.6	<0.001*		
1-year	240	43.36 ± 10.98	8.1–62.6		−16.85 (−19.34–−14.35)	<0.001*
5-year	183	43.55 ± 11.76	7.6–60.3		0.18 (−2.56–2.93)	0.990
10-year	138	43.51 ± 10.49	9.9–60.7		0.03 (−3.19–3.12)	0.990
15-year	66	41.59 ± 9.46	17.8–56.4		1.92 (−2.26–6.11)	0.720
**VR-12 mental ( $\bar{x}$ = 50)** ^c^						
Pre-op	263	43.82 ± 12.85	15.6–68.2	<0.001*		
1-year	240	51.91 ± 9.41	14.6–67.4		−8.09 (−10.61–−5.57)	<0.001*
5-year	183	51.78 ± 8.31	15.7–64.7		−0.13 (−2.90–2.64)	0.990
10-year	138	53.87 ± 9.26	26.4–70.0		2.09 (−1.09–5.28)	0.376
15-year	66	54.86 ± 9.31	18.8–71.0		0.98 (−5.21–3.24)	0.970

Diff, difference; PROM, patient-reported outcome measure; CI, confidence interval; WOMAC, Western Ontario and McMaster Universities Arthritis Index; VR-12, Veterans Rand 12-item heath survey.

a Oxford Hip Score minimum is 0 and the maximum score is 48 with poorer scores being closer to 0.

b WOMAC minimum scores are 0 with 0 being the better score.

c VR-12 ideal score is ⩾50 and acceptable is within 1 standard deviation either side.

Note: Post hoc *p*-value represents the pairwise comparison of the previous timepoint to the current timepoint.

* Significance is set at *p* < 0.05.

Publicly funded patients had statistically significantly poorer preoperative PROMs and poorer postoperative PROMs than privately funded patients across most outcomes. Mean WOMAC-pain at 10 years, VR-12-mental at 1-year, and WOMAC-motion at 1, 5, and 10 years postoperatively were still poorer in publicly funded patients, although not reaching statistical significance (*p* ⩾ 0.053) (Table 3).

**Table 3.** Male versus female patient-reported outcomes.

PROM	Male		Female		Mean diff (95% CI)	p-value
	n	Mean ± SD	n	Mean ± SD		
**Oxford (0–48)** ^a^						
Pre-op	120	19.17 ± 8.70	143	16.55 ± 8.62	−2.62 (−4.73–−0.50)	0.015*
1-year	114	41.97 ± 6.31	134	40.47 ± 7.56	−1.50 (−3.31–0.31)	0.104
5-year	83	42.77 ± 6.63	97	40.63 ± 8.02	−2.14 (−4.33–0.05)	0.055
10-year	72	42.26 ± 7.43	71	40.82 ± 8.72	−1.44 (−4.24–1.36)	0.311
**WOMAC total (0–96)** ^b^						
Pre-op	120	51.77 ± 19.20	142	57.40 ± 19.02	5.63 (0.96–10.29)	0.018*
1-year	103	10.51 ± 11.81	132	13.81 ± 13.18	3.30 (0.04–6.57)	0.047*
5-year	82	10.66 ± 13.01	97	15.32 ± 15.81	4.66 (0.34–8.98)	0.034*
10-year	63	13.63 ± 15.21	74	15.53 ± 17.27	1.72 (−3.65–7.44)	0.501
**WOMAC pain (0–20)** ^b^						
Pre-op	120	10.38 ± 3.77	142	11.56 ± 4.13	1.18 (0.21–2.15)	0.017*
1-year	103	1.46 ± 2.29	132	1.88 ± 2.62	0.42 (−0.23–1.06)	0.207
5-year	82	1.52 ± 2.69	98	2.15 ± 3.30	0.63 (−0.27–1.53)	0.168
10-year	63	2.29 ± 3.37	76	2.59 ± 3.75	0.3 (−0.90–1.51)	0.617
**WOMAC function (0–68)** ^b^						
Pre-op	120	36.92 ± 14.29	142	40.85 ± 14.21	3.93 (0.45–7.41)	0.027*
1-year	103	7.90 ± 9.02	132	10.39 ± 10.07	2.49 (−0.00–4.98)	0.050
5-year	82	7.99 ± 9.62	98	11.69 ± 12.17	3.70 (0.50–6.91)	0.023*
10-year	63	10.13 ± 11.40	75	11.51 ± 12.64	1.38 (−2.71–5.47)	0.505
**WOMAC motion (0–8)** ^b^						
Pre-op	120	4.75 ± 1.80	142	4.99 ± 1.61	0.24 (0.10–0.93)	0.015*
1-year	103	1.14 ± 1.28	132	1.54 ± 1.51	0.40 (0.03–0.77)	0.032*
5-year	82	1.15 ± 1.37	97	1.33 ± 1.40	0.18 (−0.23–0.60)	0.380
10-year	63	1.22 ± 1.46	74	1.46 ± 1.63	0.24 (−0.29–0.77)	0.376
**VR-12 physical ( $\bar{x}$ = 50)** ^c^						
Pre-op†	120	28.13 ± 8.56	143	25.16 ± 7.70	−2.97 (−4.94–−0.99)	0.003*
1-year	104	45.98 ± 9.75	136	41.36 ± 11.47	−4.62 (−7.39–−1.87)	0.001*
5-year	82	44.97 ± 11.61	101	42.39 ± 11.83	−2.58 (−6.02–0.86)	0.141
10-year	62	43.64 ± 9.93	76	43.41 ± 10.99	−0.23 (−3.79–3.34)	0.901
**VR-12 mental ( $\bar{x}$ = 50)** ^c^						
Pre-op †	120	45.59 ± 12.68	143	42.34 ± 12.86	−3.25 (−6.36–−0.13)	0.041*
1-year	104	52.85 ± 7.52	136	51.20 ± 10.61	−1.65 (−3.96–0.66)	0.160
5-year	88	53.02 ± 7.67	101	50.78 ± 8.70	−2.24 (−4.66–0.19)	0.070
10-year	61	52.67 ± 8.94	76	54.85 ± 9.46	2.18 (−0.95–5.30)	0.170

Diff, difference; PROM, patient-reported outcome measure; WOMAC, Western Ontario and McMaster Universities Arthritis Index; VR-12, Veterans Rand 12-item heath survey.

a Oxford Hip Score minimum is 0 and the maximum score is 48 with poorer scores being closer to 0.

b WOMAC minimum scores are 0 with 0 being the better score.

c VR-12 ideal score is ⩾50 and acceptable is within 1 standard deviation either side.

* Significance is set at *p* < 0.05.

Note: Post hoc *p*-value represents the pairwise comparison of the previous timepoint to the current timepoint.

**Table 4.** Public versus private patient-reported outcomes.

PROM	Public		Private		Mean diff (95% CI)	p-value
	n	Mean ± SD	n	Mean ± SD		
**Oxford (0–48)** ^a^						
Pre-op	87	10.89 ± 5.66	176	21.14 ± 7.97	10.25 (8.57–11.93)	<0.001*
1-year	73	38.60 ± 8.46	165	42.44 ± 6.05	3.84 (1.47–5.81)	0.001*
5-year	58	39.43 ± 9.11	122	42.66 ± 6.32	3.23 (0.586–5.86)	0.017*
10-year	32	37.53 ± 9.56	101	42.74 ± 7.25	5.21 (1.502–8.920)	0.006*
**WOMAC total (0–96)** ^b^						
Pre-op	86	69.72 ± 14.06	176	47.55 ± 17.19	−22.17 (−26.38–−17.97)	<0.001*
1-year	70	16.69 ± 14.91	165	10.53 ± 11.14	−6.16 (−10.093–−2.224)	0.002*
5-year	56	18.11 ± 17.09	123	10.94 ± 13.01	−7.17 (−12.27–−2.06)	0.006*
10-year	32	21.56 ± 17.20	105	12.55 ± 15.53	−9.01 (−15.37–−2.65)	0.005*
**WOMAC pain (0–20)** ^b^						
Pre-op	86	13.81 ± 3.13	176	9.66 ± 3.68	−4.151 (−5.06–−3.25)	<0.001*
1-year	70	2.54 ± 3.02	165	1.34 ± 2.13	−1.20 (−1.99–−0.42)	0.003*
5-year	57	2.63 ± 3.75	123	1.51 ± 2.59	−1.12 (−2.21–−0.03)	0.045*
10-year	32	3.41 ± 3.61	107	2.17 ± 3.53)	−1.24 (−2.65–0.18)	0.086
**WOMAC function (0–68)** ^b^						
Pre-op	86	50.07 ± 10.43	176	33.66 ± 12.86	−16.41 (−19.34–−13.48)	<0.001*
1-year	70	12.59 ± 11.15	165	7.91 ± 8.65	−4.68 (−7.64–−1.72)	0.002*
5-year	57	13.61 ± 13.12	123	8.33 ± 9.81	−5.28 (−9.16–−1.40)	0.008*
10-year	32	16.47 ± 12.76	107	9.19 ± 11.38	−7.28 (−11.95–−2.61)	0.003*
**WOMAC motion (0–8)** ^b^						
Pre-op	86	5.84 ± 1.54	176	4.23 ± 1.54	−1.61 (−2.01–−1.21)	<0.001*
1-year	70	1.56 ± 1.52	165	1.28 ± 1.38	−0.278 (−0.68–0.12)	0.173
5-year	56	1.57 ± 1.59	123	1.10 ± 1.26	−0.473 (−0.95–0.01)	0.053
10-year	32	1.69 ± 1.67	105	1.25 ± 1.51	−0.44 (−1.06–0.18)	0.162
**VR-12 physical ( $\bar{x}$ = 50)** ^c^						
Pre-op	87	23.04 ± 5.74)	176	28.23 ± 8.72	5.19 (3.42–6.97)	<0.001*
1-year	74	39.92 ± 11.06	166	44.90 ± 10.62)	4.98 (2.01–7.93)	0.001*
5-year	59	38.97 ± 13.18	124	45.72 ± 10.40	6.75 (2.88–10.63)	0.001*
10-year	32	37.19 ± 10.82	106	45.43 ± 9.65	8.24 (4.28–12.20)	<0.001*
**VR-12 mental ( $\bar{x}$ = 50)** ^c^						
Pre-op	87	34.64 ± 11.53	176	48.36 ± 10.92	13.72 (10.85–16.59)	<0.001*
1-year	74	50.27 ± 10.76	166	52.64 ± 8.68	2.37 (−0.44–5.18)	0.098
5-year	59	49.29 ± 10.26	124	52.97 ± 6.93	3.68 (0.74–6.60)	0.015*
10-year	32	48.94 ± 8.85	106	55.36 ± 8.90	6.42 (2.88–9.97)	<0.001*

Diff, difference; CI, confidence interval; PROM, patient-reported outcome measure; WOMAC, Western Ontario and McMaster Universities Arthritis Index; VR-12, Veterans Rand 12-item heath survey.

a Oxford Hip Score minimum is 0 and the maximum score is 48 with poorer scores being closer to 0.

b WOMAC minimum scores are 0 with 0 being the better score.

c VR-12 ideal score is ⩾50 and acceptable is within 1 standard deviation either side.

* Significance is set at *p* < 0.05.

Note: Post hoc *p*-value represents the pairwise comparison of the previous timepoint to the current timepoint.

1-way ANOVA revealed no statistically significant differences in any preoperative PROMs, postoperative WOMAC-pain, or postoperative VR-12-mental scores at any timepoint between ASA ratings. However, postoperative Oxford, WOMAC-function, WOMAC-motion, and VR-12-physical scores were typically poorer with higher ASA ratings at various timepoints. There were no statistically significant differences in any outcomes between ethnicities.

### PROM predictors: preoperative

No statistically significant collinearity between predictors was identified. Regression analysis indicated preoperative Oxford and WOMAC scores were negatively influenced when funding pathway was public, patients were females, and BMI was greater (Table 5). Preoperative scores for VR-12 were also negatively influenced when funding pathway was public, and patients were females.

**Table 5.** Multiple linear regression results for preoperative patient-reported outcomes.

PROM	*n*	Model *p-*value	*R²*	Estimate	*STD Error*	*p-*value
**Oxford pre-op**	263	<0.001*	0.33			
(intercept)				26.91	2.94	<0.001*
BMI				−0.25	0.10	0.015*
Sex (Male)				3.20	0.98	0.001*
Funding (Public)				−10.46	1.10	<0.001*
**WOMAC total pre-op**	262	<0.001*	0.34			
(intercept)				22.55	7.80	0.004*
BMI				0.95	0.28	<0.001*
Sex (Male)				−7.80	2.45	0.002*
Funding (Public)				22.85	2.81	<0.001*
**WOMAC pain pre-op**	262	<0.001*	0.29			
(intercept)				5.14	1.67	0.002*
BMI				0.17	0.06	0.005*
Sex (Male)				−1.46	0.52	0.006*
Funding (Public)				4.37	0.60	<0.001*
**WOMAC function pre-op**	262	<0.001*	0.33			
(intercept)				14.59	5.84	0.014*
BMI				0.73	0.21	<0.001*
Sex (Male)				−5.67	1.83	0.002*
Funding (Public)				16.76	2.11	<0.001*
**WOMAC motion pre-op**	262	<0.001*	0.24			
(intercept)				2.92	0.77	<0.001*
BMI				0.05	0.03	0.053
Sex (Male)				−0.71	0.24	0.004*
Funding (Public)				1.79	0.28	<0.001*
**VR12 physical pre-op**	263	<0.001*	0.12			
(intercept)				27.30	0.88	<0.001*
Funding (Public)				−5.82	1.33	<0.001*
Sex (Male)				2.92	1.19	0.015*
VR12 Mental pre-op	263	<0.001*	0.30			
(intercept)				47.18	1.18	<0.001*
Sex (Male)				5.50	1.60	<0.001*
Funding (Public)				−14.48	1.79	<0.001*

ASA, American Society of Anesthesiologists physical status classification; PROM, patient-reported outcome measure; STD, standard; WOMAC, Western Ontario and McMaster Universities; Arthritis Index; VR-12, Veterans Rand 12-item health survey.

* Significance set at *p* < 0.05.

### PROM predictors: postoperative

As ANOVA testing of PROMs revealed no statistically significant difference in scores from 1-year to 15 years postoperatively, 1-year scores were used as the main outcome in regression analysis given the greater number of observations. As sex, funding pathway, and BMI were significant predictors of preoperative scores, they were removed as postoperative predictors. Therefore, regression models considered preoperative score, age, ethnicity, and ASA rating. Preoperative score was a statistically significant predictor of postoperative score for all PROMs (Table 6). Increased age and ASA 3 category were additional statistically significant predictors of poorer postoperative WOMAC-function and VR-12-physical scores.

**Table 6.** Multiple linear regression results for postoperative patient-reported outcomes.

PROM	*n*	Model *p*-value	*R*²	Estimate	*STD Error*	*p*-value
**Oxford post-op**	238	<0.001*	0.07			
(intercept)				36.81	1.12	<0.001*
Oxford pre-op				0.22	0.05	<0.001*
**WOMAC total post-op**	235	<0.001*	0.15			
(intercept)				3.29	2.74	0.232
WOMAC total pre-op				−0.70	2.08	<0.001*
ASA II				−1.11	2.04	0.586
ASA III				7.66	2.85	0.008*
**WOMAC pain post-op**	235	<0.001*	0.07			
(intercept)				0.12	0.46	0.796
WOMAC pain pre-op				0.15	0.04	<0.001*
WOMAC function post-op	235	<0.001*	0.20			
(intercept)				−10.77	5.97	0.073
WOMAC function pre-op				0.18	0.04	<0.001*
Age				0.19	0.08	0.024*
ASA II				−1.40	1.59	0.380
ASA III				5.67	2.23	0.012*
**WOMAC motion post-op**	235	0.005*	0.04			
(intercept)				0.65	0.29	0.024*
WOMAC Motion pre-op				0.17	0.06	0.005*
**VR12 physical post-op**	240	<0.001*	0.28			
(intercept)				58.54	6.66	<0.001*
VR12 Physical pre-op				0.53	0.08	<0.001*
Age				−0.40	0.09	<0.001*
ASA II				−0.66	1.82	0.717
ASA III				−5.56	2.52	0.029*
**VR12 mental post-op**	240	<0.001*	0.11			
(intercept)				40.77	2.48	<0.001*
VR12 Mental pre-op				0.25	0.05	<0.001*

PROM, patient-reported outcome measure; VR-12, Veterans Rand 12-item health survey.

* Significance set at *p* < 0.05.

## Discussion

Our results indicate that primary THA with the specified cemented cup-and-stem combination for osteoarthritis improves PROMs in patients at 1-year postoperatively and that improvement is maintained in those without revision to 15 years post-surgery. Patients who were female, in the public system, and who had greater BMIs presented with poorer preoperative PROMs. Poorer preoperative PROMs negatively influenced postoperative scores across timepoints. For some outcomes, postoperative PROMs were negatively affected by higher comorbidity status (based on ASA rating) and increased age at surgery.

The substantial improvement in PROMs seen post-surgery up to 15 years with this cemented cup-and-stem combination sets a benchmark against which PROMs from other cemented or alternative implant combinations in the same or similar populations can be measured. Mean 1-, 5-, and 10-year Oxford scores were over 41, corresponding to a Kalairajah category 1 (excellent) rating.^22^ Mean 15-year Oxford scores were 39.44, corresponding to a category 2 (very good) rating.^22^ Poor 6-month Oxford scores have been identified as a risk factor for early revision within 2 years;^23^ thus, our implant combination’s Oxford score suggests low early-revision risk, which needs confirmation via survivorship analysis. The mean difference in pre- and postoperative Oxford scores (>23) is substantially higher for this implant than the estimated minimally important change value of 11,^24^ suggestive of meaningful improvements for patients.

We identified preoperative PROMs as significantly influencing postoperative PROMs, consistent with previous studies.^25,26^ Poor preoperative scores across multiple PROMs increase likelihood of patient dissatisfaction postoperatively.^26^ Our regression models revealed patients who are female, in the public system, and have higher BMIs were associated with poorer preoperative functional and mental scores, which may predispose them to postoperative dissatisfaction. Studies show that females are more likely opposed to surgery due to competing responsibilities, need more information before deciding, and are less likely to be sent to a specialist; therefore, females drop to a lower preoperative functional level than males.^27^ These observations align with our findings, wherein females’ preoperative scores were lower than males. Alongside poorer functional and mental preoperative scores, females also tend to have higher rates of depression, lower back pain, and comorbidities than males.^28^ In the TOR registry, there was a greater number of females undergoing surgery than males, consistent with national THA surgery data from NZ and other parts of the world.^4^ In the USA, not only are more females having THA surgeries,^25^ they are also more likely to be older, have higher BMIs, and need preoperative support than males.^29^ In a Korean study of over 1.15 million females, reproductive factors (e.g., later onset of menarche, longer time breastfeeding, hormone replacement treatment, and oral contraceptive use) were associated with higher risk for severe osteoarthritis and need for THA.^30^ Further investigation into sex differences in THA is required to improve patient care – particularly of females – and may need to be considered in future risk-stratification models,^29^ which should include more detailed reproductive history notetaking. Sex disparity in referral for surgery also warrants consideration.

Consistent with our observations, Greene et al.^31^ identified that patients using public funding pathways for THA report poorer preoperative and postoperative PROMs than those using private funding. Poorer public outcomes are often attributed to longer wait times; however, literature indicates the effect of increased waiting time on THA outcome is unclear or inconsistent,^32^ and that sex, age, and certain modifiable risk factors (e.g., BMI, income level, and comorbidities) impact postoperative outcomes to a greater extent.^33^ Higher BMI has also been identified as a risk factor for increased complications and poorer postoperative THA outcomes.^34^ Taken together, our findings support existing literature that women, public patients, patients with higher BMI, older patients, and patients with comorbidities (i.e., higher ASA ratings) are at greater risk of poorer postoperative THA outcomes and require greater preoperative and postoperative support. Specifically, these patients may benefit from additional mental and functional prehabilitation and rehabilitation support. Recording comorbidities in more detail than relying on ASA ratings within registry data may also help discern more specifically those conditions that may increase risk of poor THA outcomes.

Although preoperative PROMs, ASA rating, and age were identified as significant predictors of postoperative PROMs, these factors only explained between 7 and 28% of the variance (Table 5). Hence, other factors not included in our analysis likely influence the variation in postoperative scores within our population. One such factor may be smoking status. Yue et al.^35^ identified 4 studies where smoking negatively affected postoperative THA PROMs between 6 months and 1−year. Smokers are reported to exhibit lower postoperative Oxford^36^ and Harris hip^37^ scores and an increased use of painkillers and anti-inflammatories^36^ than non-smokers and ex-smokers. In smokers, PROMs were more likely to stagnate initially, and decline over time.^38^ It may be beneficial to record smoking status in future THA studies and registries.

Few THA patients in the TOR registry were Māori (4%), with a lower representation than the NZ joint registry at 7%.^4^ Considering Māori make up 29% of the Bay of Plenty region, 17% of the NZ population, and 16% of Māori are >55 years, uptake of surgery by Māori is relatively poor.^39^ Bourke et al.^40^ identified competing responsibilities (prioritising work and family), disrupted *mana*^1^ (feeling disempowered), and systemic abdication (inconsistent information/diagnosis) as contributing factors to healthcare accessibility for Māori, which may explain their under-representation as THA patients. Social deprivation and socio-economic inequities are potential sources of health inequity for Māori,^41^ which can extend to THA. Low numbers of Māori and “Other” patients made it challenging to discern differences based on ethnicity, which is further complicated due to the diversity of “Other” ethnicities.

### Limitations

Observational studies are limited in their ability to define causation; however, they can identify associations between risk factors and a response variable. Additional variables, such as smoking status, physical activity, analgesic and anti-inflammatory use, rehabilitation programmes, and specific comorbidities were not documented in the registry and therefore not analysed. Older age was identified as a predictor of poorer health scores. However, health scores also tend to deteriorate over time as age increases regardless of surgery and this was not noted most likely due to small sample sizes at 15 years. Two thirds (66%) of all surgeries within the TOR registry were privately funded, which is higher than the 2023 NZ rate of 49%.^4^ Hence, generalisation of our findings is not ensured to regions where the proportion of publicly funded surgeries is greater. Further, PROMs recorded by patients are subjective, susceptible to bias, and dependent on accurate recall.

## Conclusion

The cemented combination studied demonstrated excellent PROMs out to 15 years with the association of poorer preoperative PROMs in THAs that were publicly funded, in females and those with higher BMIs, and association of poorer preoperative PROMs, older age and ASA 3 rating variables to poorer postoperative PROMs. Patients with poorer preoperative PROMs may require additional preoperative support to improve their postoperative outcomes, encompassing both functional and mental strategies. These patients, as well as those who are older and present with higher ASA ratings, may require additional postoperative support. These associated variables, sex, ethnic disparity and accessibility to surgery need further research in NZ.

## Supplemental Material

sj-pdf-1-hpi-10.1177_11207000251371132 – Supplemental material for 15-year patient-reported outcomes of a cemented flanged cup and stem combination in primary total hip arthroplasty: a New Zealand study Supplemental material, sj-pdf-1-hpi-10.1177_11207000251371132 for 15-year patient-reported outcomes of a cemented flanged cup and stem combination in primary total hip arthroplasty: a New Zealand study by Amy Pearce, Chaitanya Joshi, Georgina Chan, Tony Lamberton, Simon MacLean, Andrew Vane and Kim Hébert-Losier in HIP International

sj-pdf-2-hpi-10.1177_11207000251371132 – Supplemental material for 15-year patient-reported outcomes of a cemented flanged cup and stem combination in primary total hip arthroplasty: a New Zealand study Supplemental material, sj-pdf-2-hpi-10.1177_11207000251371132 for 15-year patient-reported outcomes of a cemented flanged cup and stem combination in primary total hip arthroplasty: a New Zealand study by Amy Pearce, Chaitanya Joshi, Georgina Chan, Tony Lamberton, Simon MacLean, Andrew Vane and Kim Hébert-Losier in HIP International

sj-pdf-3-hpi-10.1177_11207000251371132 – Supplemental material for 15-year patient-reported outcomes of a cemented flanged cup and stem combination in primary total hip arthroplasty: a New Zealand study Supplemental material, sj-pdf-3-hpi-10.1177_11207000251371132 for 15-year patient-reported outcomes of a cemented flanged cup and stem combination in primary total hip arthroplasty: a New Zealand study by Amy Pearce, Chaitanya Joshi, Georgina Chan, Tony Lamberton, Simon MacLean, Andrew Vane and Kim Hébert-Losier in HIP International

sj-pdf-4-hpi-10.1177_11207000251371132 – Supplemental material for 15-year patient-reported outcomes of a cemented flanged cup and stem combination in primary total hip arthroplasty: a New Zealand study Supplemental material, sj-pdf-4-hpi-10.1177_11207000251371132 for 15-year patient-reported outcomes of a cemented flanged cup and stem combination in primary total hip arthroplasty: a New Zealand study by Amy Pearce, Chaitanya Joshi, Georgina Chan, Tony Lamberton, Simon MacLean, Andrew Vane and Kim Hébert-Losier in HIP International

## Notes

1. Mana- Power, authority, or prestige; *spec.* (in Polynesian and Melanesian religions) an impersonal supernatural power which can be associated with people or with objects and which can be transmitted or inherited.^42^
