# Supplementary material for: 15-year patient-reported outcomes of a cemented flanged cup and stem combination in primary total hip arthroplasty: a New Zealand study
Source: Hip Int. 2025 Oct 31;36(1):34–44. doi: 10.1177/11207000251371132 (PMC12876424; doi:10.1177/11207000251371132)
Supplement: sj-pdf-3-hpi-10.1177_11207000251371132 – Supplemental material for 15-year patient-reported outcomes of a cemented flanged cup and stem combination in primary total hip arthroplasty: a New Zealand study [file sj-pdf-3-hpi-10.1177_11207000251371132.pdf]

Supplementary table 8. Detailed revision reasons and type of revision (n = 17)

Time period	Revisions	Revision reason	(n) of each	Revision type performed
0-1 year	3	Dislocation	2	Change of femoral head
				Change of femoral head and liner
		Loosening Acetabulum (low grade infection)	1	Change of acetabulum
1-5 years	4	Periprosthetic fracture	2	Change of femoral stem
		Dislocation	1	Change of acetabulum and liner
		Pain	1	Change of acetabulum and head
5-10 years	6	Dislocation	3	Change of acetabulum, head and liner
				Change of head and liner
				Change of head
		Infection	2	Change of femur, acetabulum and head
				Change of all
		Pain	1	Change of all
10-15 years	4	Aseptic loosening- Acetabulum	2	Change of acetabulum, head and liner
		Aseptic loosening- Acetabulum and stem	1	Change of all
		Periprosthetic fracture	1	Change of femur and head
