# Supplementary material for: 15-year patient-reported outcomes of a cemented flanged cup and stem combination in primary total hip arthroplasty: a New Zealand study
Source: Hip Int. 2025 Oct 31;36(1):34–44. doi: 10.1177/11207000251371132 (PMC12876424; doi:10.1177/11207000251371132)
Supplement: sj-pdf-4-hpi-10.1177_11207000251371132 – Supplemental material for 15-year patient-reported outcomes of a cemented flanged cup and stem combination in primary total hip arthroplasty: a New Zealand study [file sj-pdf-4-hpi-10.1177_11207000251371132.pdf]

Supplemental Table 1. Descriptive statistics of preoperative patient characteristics for ALL observations.

Variable	Sample *n* = 388	Sex: Male *n* = 170 (44%)	Sex: Female *n* = 218 (56%)	Funding Pathway: Public *n* = 133 (34%)	Funding Pathway: Private *n* = 255 (66%)	Ethnicity: NZEU *n* = 350 (90%)	Ethnicity: Māori *n* = 16 (4%)	Ethnicity: Other *n* = 22 (6%)
Age†	71.30 ± 8.50	70.53 ± 8.09	71.91 ± 8.79	71.58 ± 8.54	71.16 ± 8.51	71.65 ± 8.33	67.06 ± 8.87	68.05 ± 10.25
BMI†	28.09 ± 4.78	28.21 ± 3.46	28.00 ± 5.61	28.59 ± 4.21	27.91 ± 4.97	28.03 ± 4.89	30.72 ± 2.24	27.69 ± 3.55
ASA Grade‡								
I	59(15%)	25 (15%)	34 (16%)	14 (11%)	45 (17%)	54(16%)	2 (13%)	3 (14%)
II	199 (51%)	82 (48%)	117 (54%)	57 (43%)	142 (56%)	183 (52%)	6 (37%)	10 (45%)
III	52 (14%)	26 (15%)	26 (12%)	27 (20%)	25 (10%)	46 (13%)	5 (31%)	1 (5%)
IV	1 (0%)	-	1 (0%)	1 (0%)	-	1 (0%)	-	-
Not recorded	77 (20%)	37 (22%)	40 (18%)	34 (26%)	43 (17%)	66 (19%)	3 (19%)	8(36%)

† The values are given as the mean and the standard deviations.

‡ Values are given as the number of patients, with percentage (number of patients divided by the total) in parentheses. Due to missing values, some categories do not equal the total number of patients for each column.

Abbreviations: ASA, American Society of Anesthesiologists Physical Status classification; BMI, Body Mass Index; NZEU, New Zealand European.
